# A Tripeptide Diapin Effectively Lowers Blood Glucose Levels in Male Type 2 Diabetes Mice by Increasing Blood Levels of Insulin and GLP-1

**DOI:** 10.1371/journal.pone.0083509

**Published:** 2013-12-27

**Authors:** Jifeng Zhang, Changyong Xue, Tianqing Zhu, Anuradha Vivekanandan, Subramaniam Pennathur, Zhongmin Alex Ma, Y. Eugene Chen

**Affiliations:** 1 Cardiovascular Center, Department of Internal Medicine, University of Michigan Medical Center, Ann Arbor, Michigan, United States of America; 2 Department of Internal Medicine, University of Michigan Medical Center, Ann Arbor, Michigan, United States of America; 3 Diapin Therapeutics Limited Liability Company, Ann Arbor, Michigan, United States of America; University of Toronto, Canada

## Abstract

The prevalence of type 2 diabetes (T2D) is rapidly increasing worldwide. Effective therapies, such as insulin and Glucagon-like peptide-1 (GLP-1), require injections, which are costly and result in less patient compliance. Here, we report the identification of a tripeptide with significant potential to treat T2D. The peptide, referred to as Diapin, is comprised of three natural L-amino acids, GlyGlyLeu. Glucose tolerance tests showed that oral administration of Diapin effectively lowered blood glucose after oral glucose loading in both normal C57BL/6J mice and T2D mouse models, including *KKay, db/db, ob/ob* mice, and high fat diet-induced obesity/T2D mice. In addition, Diapin treatment significantly reduced casual blood glucose in *KKay* diabetic mice in a time-dependent manner without causing hypoglycemia. Furthermore, we found that plasma GLP-1 and insulin levels in diabetic models were significantly increased with Diapin treatment compared to that in the controls. In summary, our findings establish that a peptide with minimum of three amino acids can improve glucose homeostasis and Diapin shows promise as a novel pharmaceutical agent to treat patients with T2D through its dual effects on GLP-1 and insulin secretion.

## Introduction

Type 2 diabetes (T2D) is one of the most prevalent human metabolic diseases and has rapidly emerged as a global health care problem reaching epidemic proportions in recent years [1]. T2D is characterized by hyperglycemia resulting from insulin resistance and relative deficiency of insulin due to the reduction of functional β-cell mass [2]. Current therapies for T2D include increasing plasma insulin levels, improving insulin sensitivity of tissues, and reducing carbohydrate absorption from the gastrointestinal tract [3]. Insulin, produced by β-cells of the pancreas, is a key hormone in regulating of carbohydrate and fat metabolism in the body. Increase of insulin levels, either by direct insulin administration or by agents that promote insulin secretion and preserve functional β-cell mass, is a key goal in the treatment of T2D. Glucagon-like peptide-1 (GLP-1), mainly produced by the intestinal L-cell, stimulates glucose-dependent insulin secretion and protects β-cells [4]–[6]. The therapeutic insulin and GLP-1, however, require injections, which are costly and inconvenient, resulting in less patient compliance.

The success of incretin-based therapies for T2D [7] and recent advances in our understanding of mechanisms for nutrient sensing [8]–[10] suggest that nutrient-based agents may hold potential for future anti-T2D drug development. Carbohydrates and fatty acids are the main sources of energy in the diet and also act as signaling molecules in various cellular processes, including stimulating insulin secretion and GLP-1 release [11], [12]. While glucose-stimulated insulin secretion and GLP-1 release are severely impaired in T2D [13], [14], the fatty acid receptors, a group of G-protein coupled receptors, such as GPR40 and GPR120 that are activated by medium- and long-chain FFAs, have been suggested as potential drug targets for T2D [15].

Proteins are essential for the growth and repair of tissues. Early studies reported that the combined intake of carbohydrate and protein have stimulatory effect on plasma insulin release [16], [17], which has been confirmed in both healthy subjects [18] and in patients with T2D [19]–[21]. Recently, additional studies have reported that ingestion of protein hydrolysates with carbohydrates improve plasma glucose disposal and reduce postprandial blood glucose in T2D patients [22], [23]. Importantly, this protein hydrolysate diet improved daily blood glucose homeostasis in long-standing T2D patients [23], [24]. It appears that protein hydrolysates can augment insulin secretion [23], [25], [26] and stimulate GLP-1 release in the rat colon fraction and in rodent and human L-cell lines [27]–[29]. Indeed, it has been reported that a protein preload in T2D patients stimulates insulin and incretin hormone secretion and slows gastric emptying, leading to marked reduction in postprandial glycemia [30].

Dietary proteins must be digested into amino acids or di- and tripeptides and then absorbed in small intestine [31]. Amino acids are absorbed by at least four sodium-dependent amino acid transporters located on the lumenal plasma membrane of the absorptive cell [32], while di- and tripeptides are absorbed into the small intestinal epithelial cells by co-transporting with H^+^ ions via a transporter called PEPT1 [33], [34].

In light of the fact that protein hydrolysates have insulinotropic effect in T2D patients and stimulate GLP-1 secretion in the isolated colon from rats and L-cell lines, we hypothesized that small peptides, such as di- or tripeptides from protein hydrolysates may be useful for treatment T2D. In this study, we screened many di and tripeptides and identified a tripeptide, referred to as Diapin, with significant potential to treat T2D. Our findings establish that tripeptides may be the active components in protein hydrolysates responsible for GLP-1 release and enhanced insulin secretion.

## Materials and Methods

### Animals

Male C57BL/6J mice (stock number 000664), *ob/ob* obese mice (B6.V-Lep^ob^/J, stock number 000632), *db/db* diabetic mice (BKS.Cg-m +/+ Lepr^db^/J, stock number 000697), *KKay* mice (KK.Cg-*Ay*/J, stock number 002468), were purchased from Jackson Laboratories (Bar Harbor, MA) and housed in a temperature-controlled animal facility with a 12 h:12 h light-dark cycle and free access to water and rodent chow unless otherwise indicated. The study protocol was approved by the University of Michigan Committee on Use and Care of Animals.

### Peptide Synthesis

Diapin (GlyGlyLeu) and other peptides used in this study were synthesized by CS Bio Co. (Menlo Park, CA). Diapin was dissolved in 1X PBS buffer before all experiments. Leucine and Glycine were purchased from Sigma (St. Louis, MO).

### Oral Glucose Tolerance Test (OGTT) or Intraperitoneal Glucose Tolerance Test (IPGTT)

The OGTT and IPGTT were performed as described previously [35]. In brief, the mice were fasted for 16 hours with free access to water and randomly divided into the control and treated groups. For OGTT, glucose at the dose of 2 mg/g body weight (bw) plus vehicle or Diapin (1 mg/g, bw) plus glucose (2 mg/g, bw) were delivered into the stomach of mice by a gavage needle (20-gauge, 38 mm long curved, with a 21/4 mm ball end), respectively. For IPTGG, after oral loading of Diapin by gavage, glucose (2 mg/g, bw) was injected into the intraperitoneal cavity. The blood glucose levels were measured by the FreeStyle glucose meter (Abbott) through tail vein blood sample at 0, 30, 60, 90, and 120 min.

### Plasma GLP-1 and Insulin Measurement

Blood was collected from mice 30 min after administration of Diapin (1 mg/g, bw) and glucose (2 mg/g, bw) and the GLP-1 level in the plasma was measured using a total GLP-1 (7–36 and 9–36) ELISA kit (Alpco. Salem, NH) and plasma insulin was measured using an ultrasensitive mouse insulin ELISA kit (Crystal Chem, Inc.) following manufacturer’s recommendation.

### Cell Culture and Treatment

The INS-1 cells [36]–[38] were cultured in RPMI 1640 medium (Invitrogen) containing 10% FBS, 10 mM HEPES buffer, 2 mM glutamine, 1 mM sodium pyruvate, 50 mM β-mercaptoethanol, and 0.1% (wt/vol) of penicillin in a 5% CO_2_ in air atmosphere at 37°C [36]–[38]. The STC-1 cells [27], [39], [40] were cultured in Dullbecco’s modified Eagle’s medium (DMEM) (25 mM glucose) (Invitrogen) containing 10% horse serum and 2% FBS with penicillin (10 U/ml) and streptomycin (10 µg/ml) [41]. For insulin secretion assay, the INS-1 cells were washed in the Krebs-Ringer bicarbonate (KRB) buffer and incubated in KRB buffer containing 3 mM glucose for 60 min. The medium was then removed and replaced with KRB buffer containing various concentration of Diapin at 37°C for 60 min. The supernatant in each well was collected for insulin measurement. For GLP-1 secretion assay, the STC-1 cells were treated with or without Diapin in the DMEM (without glucose and serum) supplemented with 0.1% BSA for 2 hours and the supernatants were collected for GLP-1 measurement.

### Plasma Diapin Measurement

To monitor the Diapin levels in the blood after oral administration, the blood samples were collected at the different time point as indicated. The plasma samples (10 ml) were diluted with 90 ml pure water, spiked with a known concentration of U-^13^C_6_^15^N_1_-Diapin (10 pmol/sample), and concentrated using 3 kDa cut off filters. The filtrate was dried at 60°C using vacuum concentrator. The dried samples were reconstituted in 0.1% formic acid and injected into the mass spectrometer. Then the Diapin levels were quantified by liquid chromatography electrospray ionization tandem mass spectrometry (LC/ESI/MS/MS). Isotope-labeled and native standard Diapin were synthesized by CS Bio Co (Menlo Park, CA, USA), with glycine labeled with ^13^C and ^15^N with the labeled peptide 4 a.m.u higher mass than native peptide. Native and labeled synthetic Diapin were reconstituted in 50% methanol. Flow injection analysis (FIA) was used to optimize the fragmentor voltage and collision energy determined by the intensity of precursor ions and product ions, respectively. The samples were subjected to reverse phase LC with a C-18 column (Agilent Technologies, New Castle, DE). Diapin was separated using 0.1% formic acid (Solvent A) and 0.1% formic acid in acetonitrile (Solvent B). The gradient was 0–20% Solvent B for 1.5 min, 20–95% Solvent B for 4.5 min, 95% Solvent B for 1.5 min and the column was re-equilibrated with solvent A for 4 min at a flow rate of 0.6 mL/min. The effluent was subjected to ESI/MS/MS with an Agilent 6410 Triple Quadrupole MS system in positive-ion mode. The MS parameters were as follows: delta EMV +1000, desolvation gas 350°C, capillary voltage 4000 V, nebulizer pressure 45 psi and the desolvation gas flow rate 11 L/min. The peptide was quantified by comparing peak areas of the native and labeled peptides by utilizing Agilent Mass Hunter Quantitative Analysis Software.

### Statistical Analysis

Data are expressed as means ± SD. Statistical analysis for comparison between two groups was performed by the unpaired Student’s t-test was used. For multiple comparisons and among 3 groups and mores, analysis was performed by 1-way ANOVA of variance. At least three independent experiments were performed for the presented data. A value of *p*<0.05 was considered statistically significant.

## Results

### Diapin Lowers Blood Glucose Level in C57BL/6J Mice after Glucose Administration

**Figure 1:**
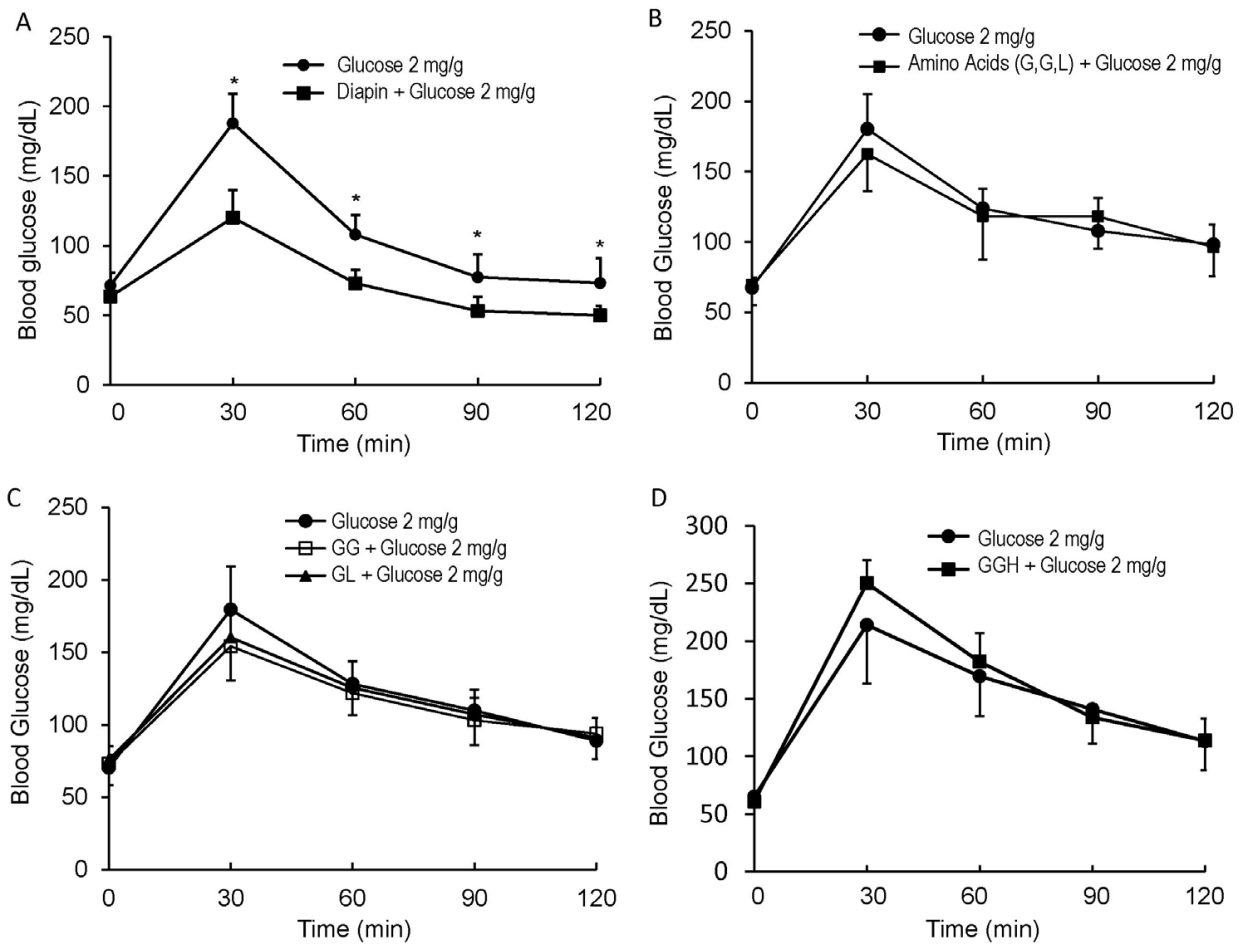
Effect of Diapin on blood glucose levels in C57BL/6J mice during OGTT. **A.** Effect of Diapin on blood glucose. The mice were fasted overnight and orally loaded with glucose (2 mg/g, bw) in the control (n = 10) and glucose (2 mg/g) plus Diapin (4 µmol/g, equal to 1 mg/g) in the treated group (n = 10). **B.** Effect of the amino acids of Diapin on blood glucose. In the treated group (n = 10), the mice were loaded with glucose and mixture of amino acids of Diapin (G, G, L; 4 µmol/g for each amino acid). **C.** Effect of the dipeptides on blood glucose. In the treated groups, the mice were loaded with glucose and dipeptides (GG or GL, 4 µmol/g). **D.** Effect of a control peptide on blood glucose. In the treated group (n = 10), the mice were loaded with glucose and a tripeptide (4 µmol/g). Blood glucose levels were measured every 30 min after glucose loading. *P<0.05, compared with the controls.

We screened many di- and tripeptides by oral administration of peptides to C57BL/6J mice and found that tripeptides were, in general, more effective than dipeptides in lowering glucose levels during GTT. Among several tripeptides that affected glucose levels, we identified one, referred as Diapin that was more effective than the others in lowering blood glucose level in C57BL/6J mice after oral loading of glucose. To rigorously determine the effect of Diapin on lowering glucose levels *in vivo*, we performed OGTT in adult male C57BL/6J mice. The fasted mice were given glucose or glucose plus Diapin by gavage and blood glucose levels were measured. As shown in Fig. 1A, Diapin significantly lowered the blood glucose levels in C57BL/6J mice. However, none of the mixture of amino acids comprising Diapin (Fig. 1B), or dipeptides (Fig. 1C), or another tripeptide (Fig. 1D) had a significant effect on glucose levels during OGTT. Although we have not examined all possible combinations of tripeptides, our finding suggests that Diapin, but not all tripeptides, can effectively lower blood glucose in mice.

**Figure 2:**
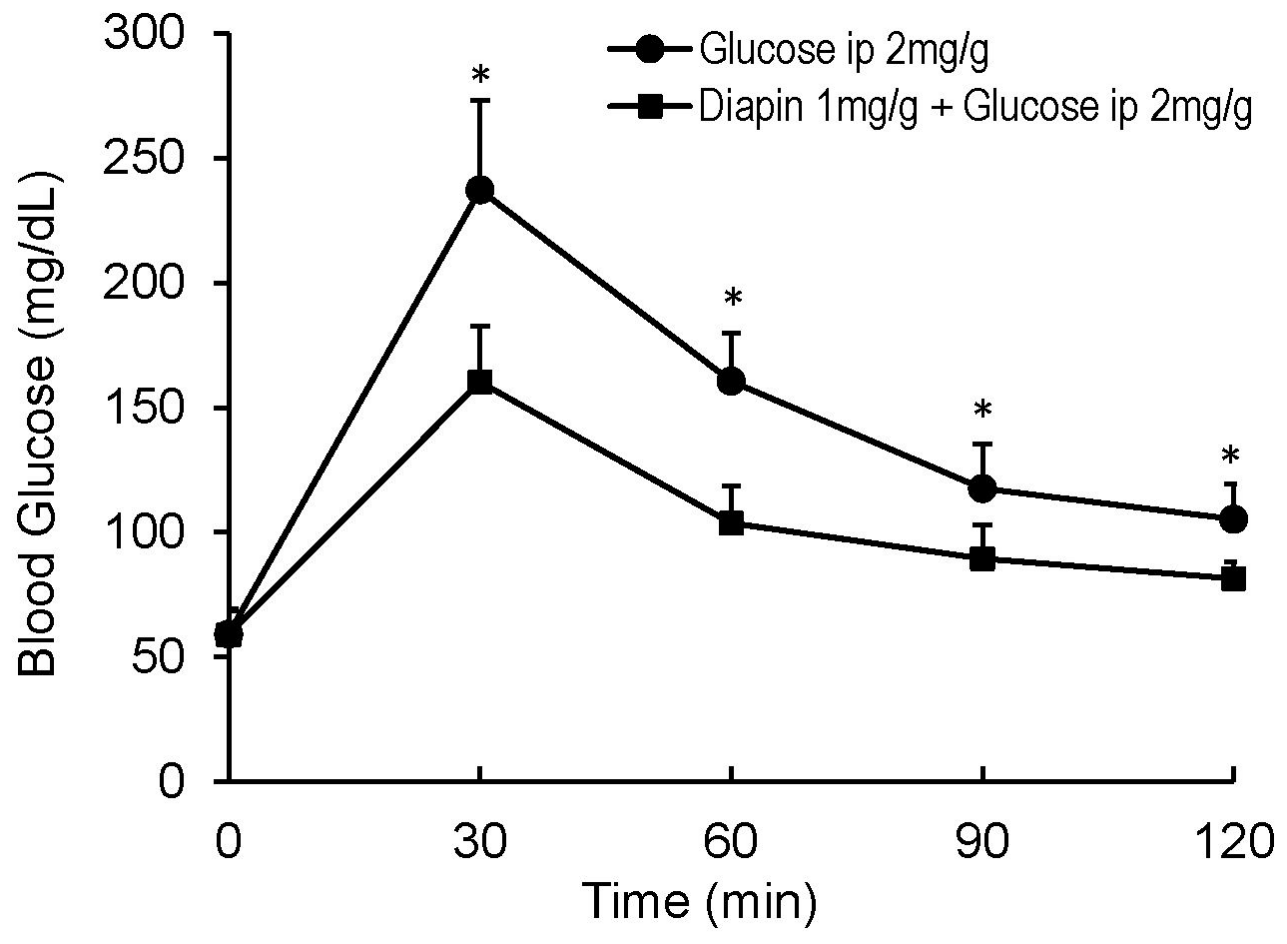
Effect of Diapin on blood glucose in C57BL/6J mice during IPGTT. The mice were orally given vehicle in the control group (n = 10) and Diapin (1 mg/g, n = 10) in the treated group and followed by IPGTT. *P<0.05, compared with the controls.

We next performed an IPGTT to determine whether the glucose lowering effect of Diapin can be attributed to its inhibition of glucose absorption in gastrointestinal track. The fasted mice were given Diapin by gavage first, followed by intraperitoneal glucose loading. Similar to the results in the OGTT, IPGTT demonstrated that Diapin significantly lowered blood glucose levels in C57BL/6J mice at all of the time points (Fig. 2), indicating that Diapin does not act by inhibiting absorption of glucose in gastrointestinal track.

### Diapin Lowers Blood Glucose Level in Diabetic Mice after Oral Glucose Administration

**Figure 3:**
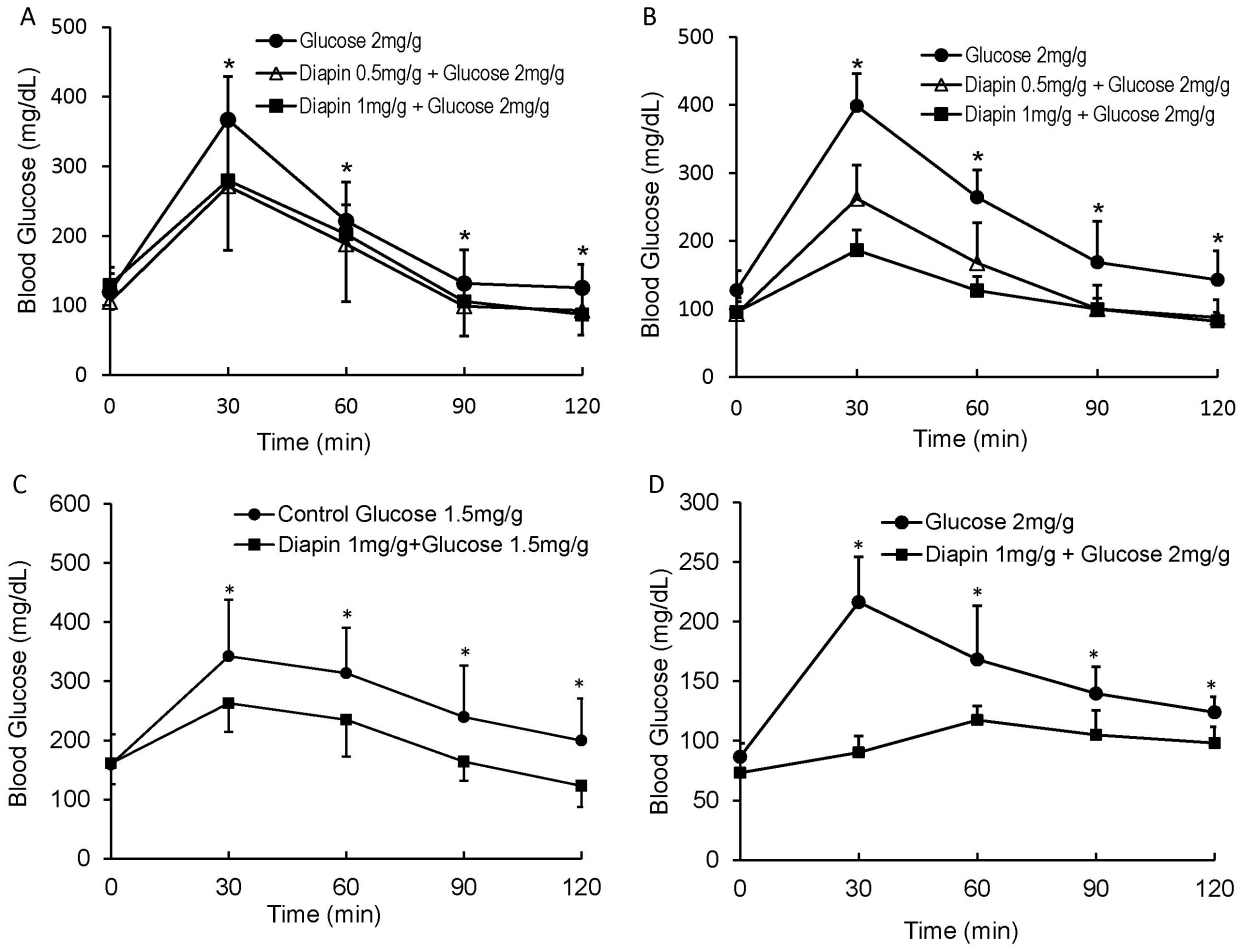
Effect of Diapin on blood glucose levels in diabetic mice. After oral loading of Diapin (Δ, 0.5 mg/g; ▪, 1 mg/g) by gavage, OGTT was performed in male (**A**) *ob/ob* (n = 10), (**B**) *db/db* (n = 10) diabetic mice, (**C**) *KKay* (n = 9), and (**D**) the high fat diet-induced obesity mice (n = 10), in which the wild type male C57BL/6J mice were fed with high fat diet for ten weeks. *p<0.05, compared with the controls.

We next determined whether Diapin was capable of lowering blood glucose in several genetic obesity/T2D mouse models. Male *ob/ob* (Fig. 3A), *db/db* (Fig. 3B), and *KKay* (Fig. 3C) diabetic mice were randomly divided into the control and the treatment groups and subjected to an OGTT. As shown in Fig. 3, Diapin at the concentration of 1 mg/g significantly lowered blood glucose levels in each of these models. In addition, we also employed the high-fat diet induced obesity/T2D in C57BL/6J mice (Fig. 3D). For this model, wild type male C57BL/6J mice were fed with high fat diet (Research Diets Inc. Cat#: D12492) for ten weeks to induce obesity with insulin resistance. Similar to the results observed in the genetic obesity/T2D mouse models, Diapin significantly lowered blood glucose levels in the high-fat diet induced obesity/T2D mice (Fig. 3D). Taken together, these results demonstrate that Diapin treatment can effectively reduce hyperglycemia in obesity/T2D mice.

### Diapin Decreases Casual Blood Glucose Levels in Diabetic Mice

**Figure 4:**
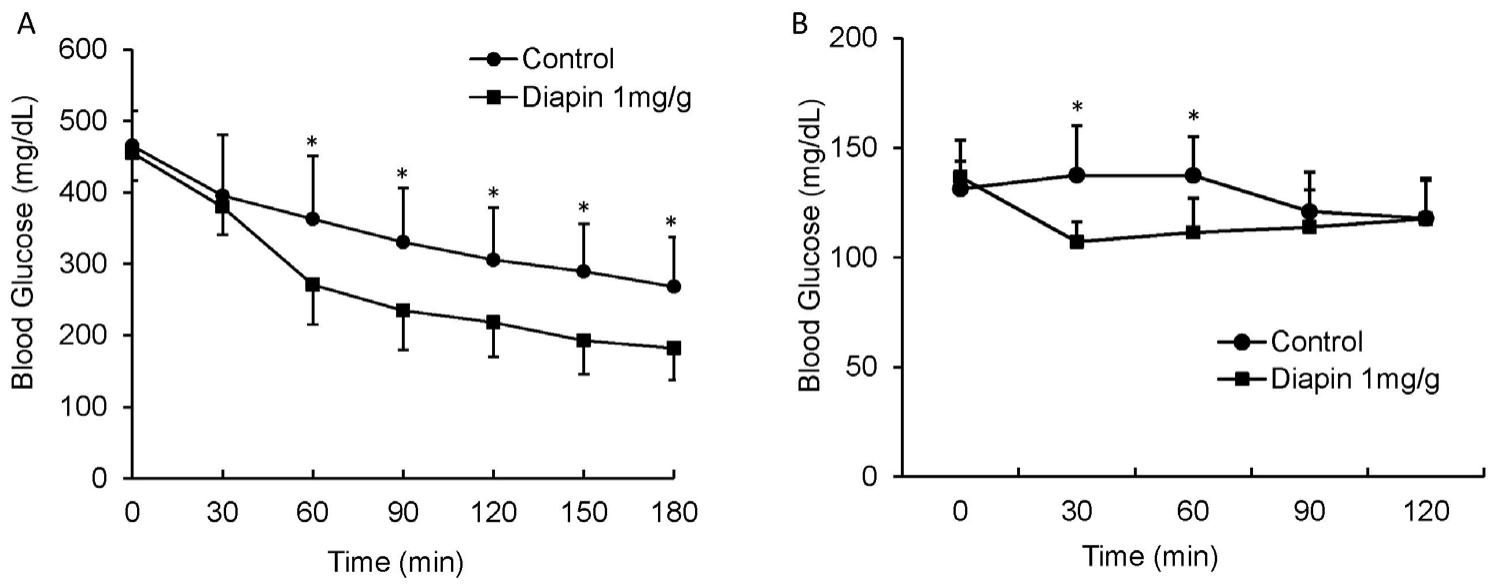
Effect of Diapin on non-fasting blood glucose levels in *KKay* diabetic mice. (A) Male *KKay* diabetic mice and (**B**) adult male C57BL/6J mice under non-fasting condition were given either vehicle in control groups or Diapin (1 mg/g bw, n = 9/group) in the treated groups. After Diapin loading, the blood glucose levels were measured every 30 min. *P<0.05, compared with the controls.

We further determined the effect of Diapin on postprandial glucose levels in *KKay* diabetic mice. The *KKay* mouse was used because it is a polygenic model of obesity and T2D with severe glucose intolerance, insulin resistance, islet hypertrophy and hyperplasia, elevated HbA1c, microalbuminuria, and developed diabetic glomerular nephritis and arteriosclerosis in males, which are more similar to the pathologic phenotypes of T2D in humans [42]–[44]. Under non-fasting condition, male *KKay* mice were given either water or Diapin and the blood glucose was measured. As shown in Fig. 4A, a significant decrease in casual blood glucose levels was observed in *KKay* diabetic mice after 60 min of Diapin treatment. However, in the normal C57BL/6J mice, although Diapin treatment decreased the casual blood glucose levels at 30 and 60 min, the postprandial glucose levels remained in normal range (Fig. 4B), indicating that Diapin does not cause hypoglycemia even under non-insulin resistance conditions. Together, these results suggest that Diapin can effectively lower blood glucose in the subjects with T2D without risk of hypoglycemia.

### Diapin Lowers Blood Glucose Levels in Diabetic Mice with Time

**Figure 5:**
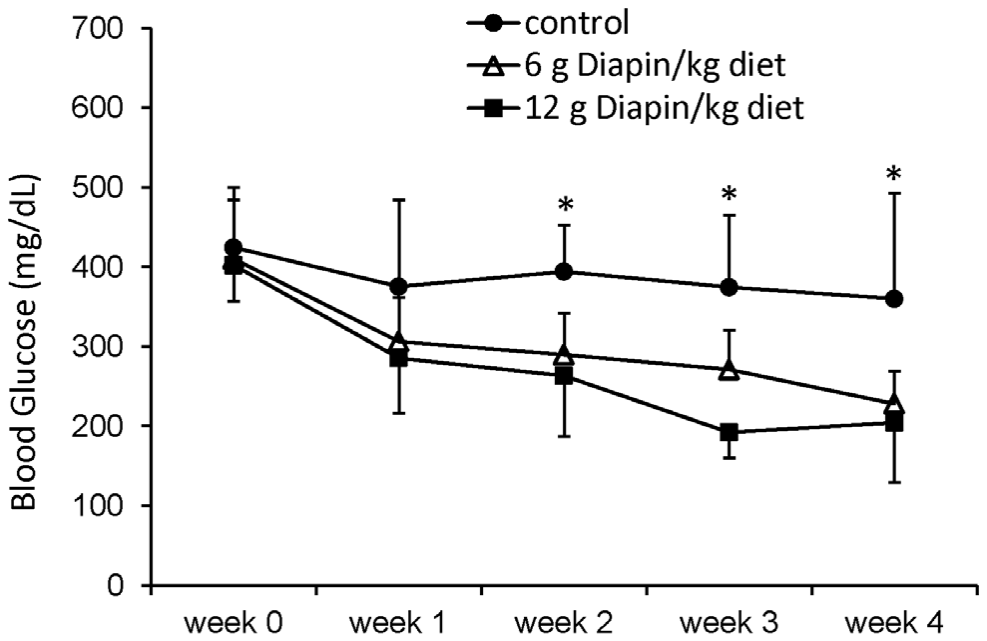
Time dependent effect of Diapin on blood glucose level in *KKay* diabetic mice. Male *KKay* diabetic mice were randomly divided into control and treated groups. In control group (•), the mice were fed with regular chaw diet. The mice in the treated groups were fed with regular chow diet containing 6 (Δ) and 12 g/kg Diapin (▪), respectively. The casual blood glucose levels were monitored weekly. n = 10, *p<0.05, compared with the control.

To determine the long-term effect of Diapin on blood glucose levels in T2D, male *KKay* diabetic mice were randomly divided into the control group, in which mice were fed with regular chow diet, and the treated group, in which mice were fed with regular chow diet containing Diapin (6 g or 12 g/kg). The blood glucose levels were monitored weekly and the results showed that Diapin at 6 g/kg diet decreased the blood glucose level of *KKay* mice in a time-dependent manner (Fig. 5).

### Diapin Elevates Plasma Insulin Levels in Diabetic Mice

**Figure 6:**
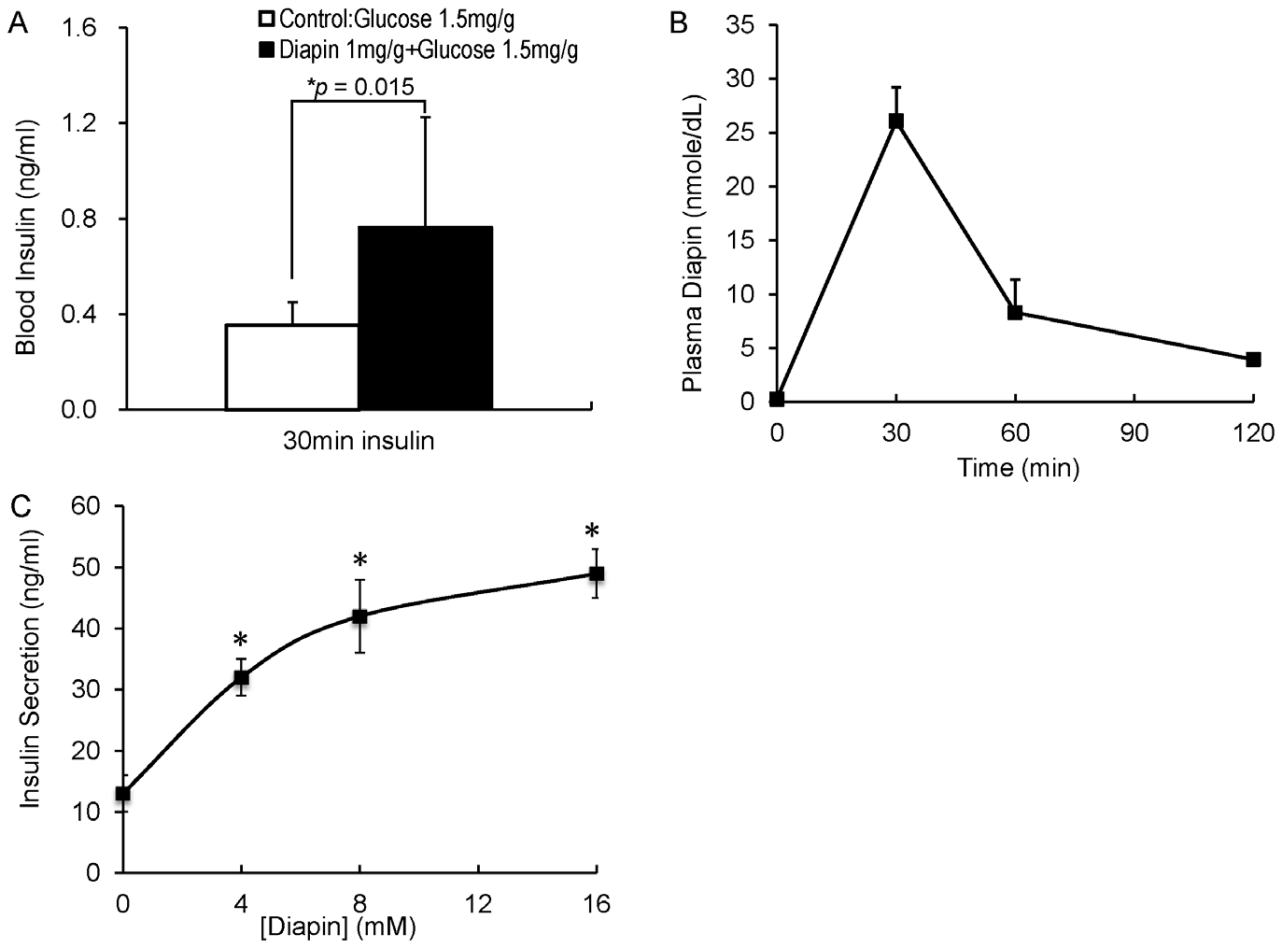
Effect of Diapin on plasma insulin levels in *KKay* diabetic mice. **A.** Insulin levels in mouse plasma. The fasted male *KKay* mice were randomly divided into two groups (n = 11/group) and orally given glucose or glucose plus Diapin, respectively. Blood samples were collected 30 min after administration of glucose and plasma insulin levels were measured. **B.** Diapin concentration in mouse plasma. The fasted male C57BL/6J mice were orally loaded with Diapin at 1 mg/g bw plus glucose 2 mg/g. Blood samples were collected at different time points and Diapin in plasma was measured by LC-MS/MS system (n = 5). **C.** Insulin secretion by INS-1 cells. INS-1 cells were treated with various concentration of Diapin for 1 hour and the supernatants were collected for insulin measurement. *P<0.05, compared with that in the absence of Diapin.

To uncover the possible mechanism underlying the glucose lowering effect of Diapin, we first determined whether Diapin treatment increases blood levels of insulin, a key hormone for control blood glucose. The fasted *KKay* diabetic mice were orally administered glucose in the control group or glucose plus Diapin in the Diapin-treated group and the blood samples were collected at 30 min for insulin measurement. As shown in Fig. 6A, plasma insulin levels in the Diapin-treated group were significantly higher than that in the control group. If Diapin can directly stimulate insulin secretion from pancreatic β-cells, it must be absorbed into the blood stream. We then determined Diapin concentration in mouse plasma after oral administration of Diapin. Fasted adult male C57BL/6J mice were orally loaded with Diapin and glucose and the blood samples were collected. Measurement of Diapin in plasma by LC-MS/MS showed that Diapin were highest at 30 min and rapidly diminished thereafter (Fig. 6B). To determine whether Diapin can directly stimulate insulin secretion, the rat β-cell line INS-1 cells were treated with Diapin. We found that Diapin stimulated insulin secretion from INS-1 cells in a concentration dependent manner (Fig. 6C). Taken together, the results suggest that Diapin is capable of entering blood stream to stimulate insulin release from pancreatic β-cells.

### Diapin Elevates Plasma GLP-1 Levels in Diabetic Mice

**Figure 7:**
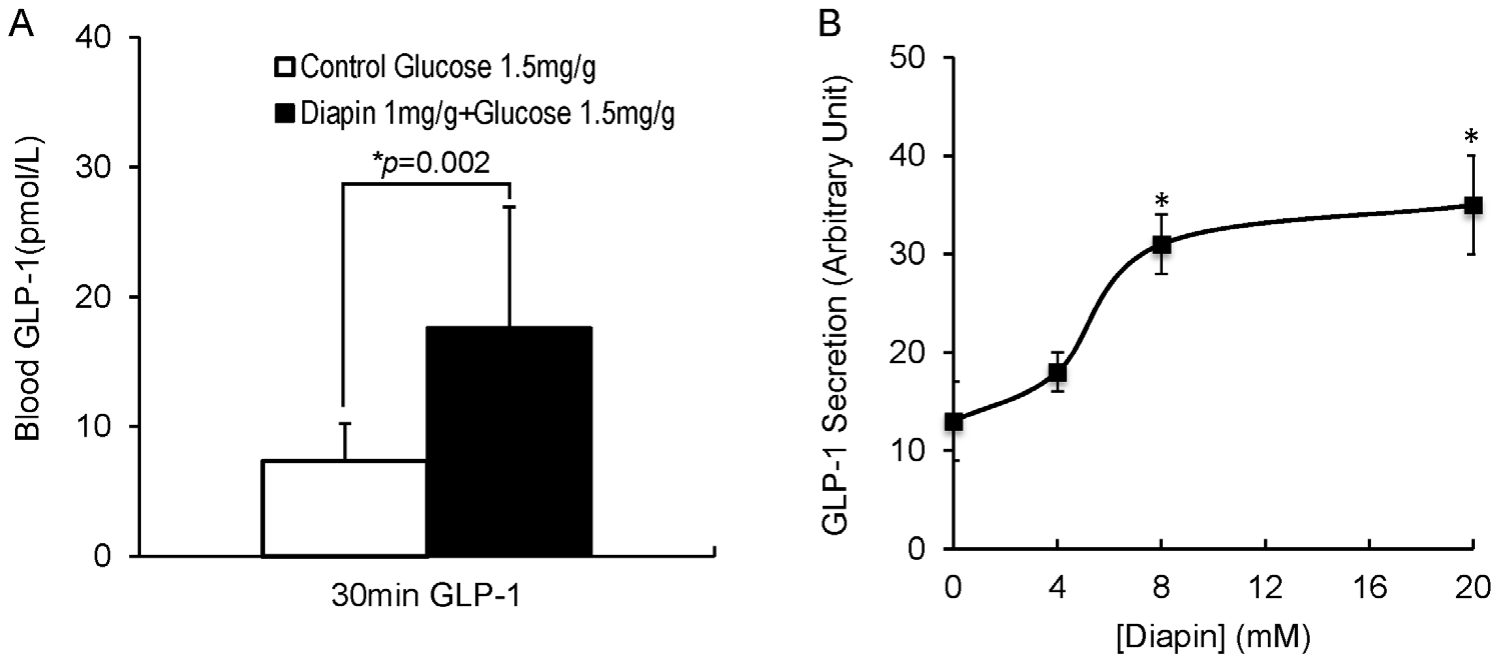
Effect of Diapin on plasma GLP-1 levels in *KKay* diabetic mice. **A.** GLP-1 levels in mouse plasma. The male *KKay* mice (n = 10/group) were treated as in Fig. 6A. Blood samples were collected 30 min after administration of glucose and plasma GLP-1 levels were measured. **B.** GLP-1 secretion by STC-1 cells. STC-1 cells were treated with various concentration of Diapin for 2 hours and the supernatants were collected for GLP-1 measurement. *P<0.05, compared with that in the absence of Diapin.

It has been reported that protein hydrolysates stimulate GLP-1 release in rat distal intestinal fraction and in rodent and human L-cell lines [27]–[29]. To determine whether oral administration of Diapin can stimulate GLP-1 secretion in the gastrointestinal tract, fasted *KKay* diabetic mice were orally given glucose in the control group or glucose plus Diapin in the Diapin-treated group and blood samples were collected at 30 min for GLP-1 measurement. As shown in Fig. 7A, total GLP-1 levels in the plasma of the Diapin-treated group were 2.4 times higher than that in the control group. We then determined whether Diapin can stimulate GLP-1 secretion from a mouse enteroendocrine L-cell line STC-1 cell and demonstrated that this indeed the case (Fig. 7B). It is well known that GLP-1 plays key role in controlling postprandial glucose levels. Together with our OGTT data in diabetic mice (Fig. 3), in which the postprandial blood glucose levels in the Diapin-treated groups were significantly lower than those in the control groups, suggesting that Diapin lowers the blood glucose levels in mice, at least in part, through stimulating GLP-1 release from enteroendocrine L-cells in gastrointestinal tract. This should further enhance insulin secretion from pancreatic islet β-cells.

## Discussion

In this study, we report the identification of a tripeptide, referred to as Diapin, with a significant potential to treat T2D. We demonstrated that oral administration of Diapin effectively lowered blood glucose levels in several genetic T2D mouse models including *KKay*, *db/db,* and *ob/ob* mice and in high-fat-diet induced obesity/T2D mice. Moreover, Diapin lowered casual glucose levels in mice in a time-dependent manner. Diapin was also capable of elevating the blood levels of both insulin and GLP-1, two hormones that are essential for regulating glucose homeostasis. Diapin is comprised of naturally occurring L-amino acids and, therefore, it is unlikely to have significant side effects. In addition, Diapin is taken orally, which is convenient for patients with T2D. Finally oral administration of Diapin apparently has no propensity to cause hypoglycemia. Thus, these advantages suggest that development of Diapin therapy holds potential as a treatment for T2D.

It is a long-time observation that protein hydrolysates have insulinotropic action, particularly in human patients with T2D [23], [25], [26], and stimulate GLP-1 release [27]–[29]. Protein hydrolysates are a mixture of heterogeneous peptides. We ask the question what are the minimum amino acid requirements and the possible sequences for the peptides from protein hydrolysates are in order to have such stimulatory effects on insulin and GLP-1 in T2D patients. We found that a tripeptide can stimulate GLP-1 release and enhance insulin secretion *in vivo*, although we have not examined all possible combinations of tripeptides. The identification of such peptide paves the way for our understanding the molecular mechanism, by which protein hydrolysates stimulate GLP-1 release and enhanced insulin secretion *in vivo*.

Increasing blood insulin levels in response to glucose intake is an important therapeutic strategy in the treatment of T2D, which can be achieved either by insulin therapy or agents that promote endogenous insulin secretion. Preserving functional β-cell mass and stimulating endogenous insulin secretion without causing hypoglycemia are the goals in the development of novel anti-diabetic drugs. Recently, GLP-1-based drugs, including GLP-1 receptor agonists and dipeptidyl peptidase 4 (DPP-4) inhibitors, are routinely used to treat T2D [7]. GLP-1-based therapies have shown the ability to stimulate glucose-dependent insulin secretion, suppress glucagon secretion, inhibit gastric emptying, reduce appetite and food intake, and possibly enhance β-cell survival in islets [4]–[6]. Although GLP-1 analog is an effective therapy for T2D with multiple advantages [45], its market share is very low with 2%, likely due to their injectable nature (According to National Health and Nutrition Examination Survey-2010). DPP-4 inhibitors are oral anti-diabetic drugs by inhibiting activity of DPP-4, an enzyme that rapidly inactivates GLP-1, and thereby prevent GLP-1 breakdown. Therefore, they share the advantages of GLP-1 analog therapy. However, DPP-4 inhibitors are less effective than GLP-1 receptor agonists for reducing HbA(1c) and body weight [46] and their side effects are emerging [47].

Here we reported that orally taken Diapin can effectively lower blood glucose levels, particularly postprandial blood glucose, by increasing the GLP-1 levels in diabetic mouse models, suggesting that Diapin therapy shares the advantages of GLP-1. Indeed, we found that oral administration of Diapin significantly reduced the casual blood glucose levels in diabetic mice without causing hypoglycemia even under non-insulin resistance states, suggesting that Diapin therapy has little risk of hypoglycemia.

Metformin is the first-line drug of choice for the treatment of T2D, in particular in overweight and obese and those with normal kidney function [48]. However, metformin alone cannot stop the progressive decline in β-cell function in patients with T2D, which ultimately leads to the need for insulin therapy [49]. Recent clinic studies showed that combined therapies of metformin with GLP-1-based therapies have better clinical outcome in terms of reducing HbA(1c) and body weight and risk of hypoglycemia [47]. Because of injectable nature of GLP-1 analog therapy and site effects of DPP-4 inhibitor therapy, it is possible that combined therapy of metformin with Diapin may provide an alternative therapy in future.

**Figure 8:**
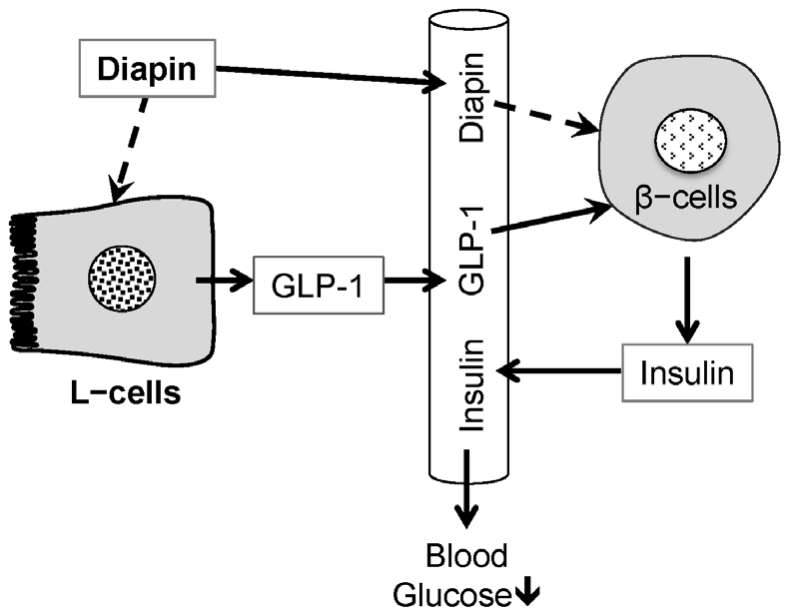
Proposed mechanism of action for Diapin to lower blood glucose in T2D. Diapin is a tripeptide that has potential to treat T2D. Oral administration of Diapin may directly act on enteroendocrine L-cells in gastrointestinal tract to stimulate GLP-1 secretion (dashed line: unknown mechanisms), which in turn stimulates the glucose-dependent insulin secretion. In addition, Diapin in the blood stream may also act on pancreatic β-cells to stimulate insulin secretion (dashed line: unknown mechanisms). This dual effect leads to the elevation of insulin and the effective control of blood glucose in T2D.

In summary, Diapin is a tripeptide that has potential to treat T2D by oral administration. We have proposed the mechanism of action for Diapin to lower blood glucose in T2D as elucidated in Fig. 8. Diapin may directly act on enteroendocrine L-cells in gastrointestinal tract to stimulate GLP-1 secretion, which in turn stimulates the glucose-dependent insulin secretion. In addition, Diapin in the blood stream may also act on pancreatic β-cells to stimulate insulin secretion. This dual effect leads to the elevation of insulin and the effective control of blood glucose in T2D. Diapin therapy may share advantages of GLP-1 therapies without causing hypoglycemia, although further studies are needed to verify its clinical outcomes in human.
